# Factors affecting insulation failure in reusable surgical devices

**DOI:** 10.1038/s41598-023-41059-8

**Published:** 2023-08-22

**Authors:** Takahiro Homma, Hirofumi Uehara, Hisashi Saji

**Affiliations:** 1 Division of Thoracic Surgery, Kurobe City Hospital, Toyama, Japan; 2 Division of Thoracic Surgery, University of Toyama, Toyama, Japan; 3 Department of Chest Surgery, St. Marianna University School of Medicine, 2-16-1 Sugao, Kawasaki, Kanagawa 216-8511 Japan; 4 Central Sterile Supply Department, Toyama University Hospital, Toyama, Japan

**Keywords:** Medical research, Risk factors

## Abstract

The purpose of this study was to investigate the associated factors of insulation failure (IF) in reusable endoscopic instruments. The insulation coating of reusable endoscopic instruments underwent routine visual checks, hand washing to remove visible stains, and mechanized sterilization. We recorded the cleaning number and usage period of all instruments. The instruments were tested for IF using a detector. IF was found in eight of 69 devices (11.6%). Examining by clinical specialty, we found IF in 4 of 28 gastrointestinal (14.3%), 3 of 20 gynecological (15.0%), 1 of 12 urological (8.3%), and none of the nine thoracic devices. The median distance from the tip to the damaged part was 5 cm (3–5 cm). In the IF and the intact groups, the period of use [7 years (6–8) versus 7 years (4–8), P = 0.90] and the number of cleanings [281 (261–323) versus 261 (179–320), P = 0.27] were not significantly different. The IF group included products of three different companies; however, six of the eight (75.0%) were from the same company. Cleaning methods and usage period have a lower impact on IF. The use of reusable forceps as a monopolar device was found to pose a higher risk, requiring regular assessments.

## Introduction

**Figure 1:**
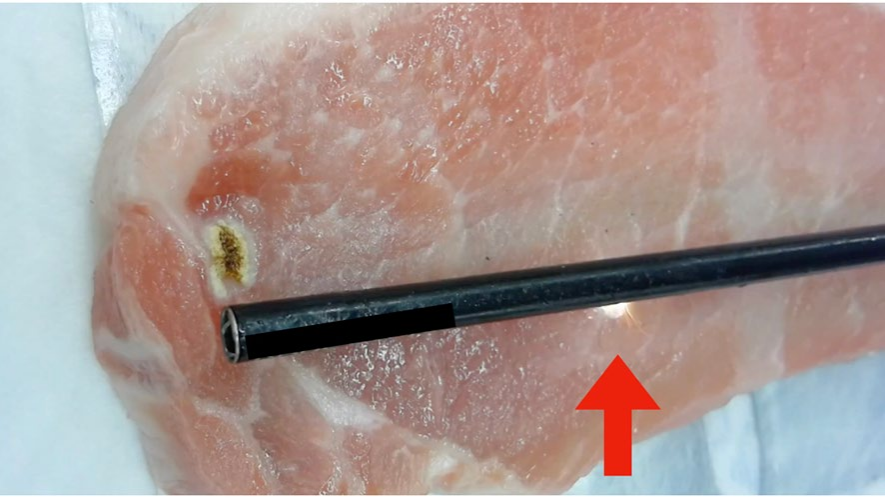
Energization experiment using an instrument with insulation failure. This photo shows burning except at the tip (red arrow).

Electrical devices have dramatically contributed to the evolution and safety of surgical treatment regardless of procedures or approaches. While they improve the quality and safety of surgery, their misuse can put patients at risk. Various adverse events caused by electrical devices have been reported^1–5^. Insulation failure (IF) is one of the causes of adverse events^6,7^. Insulation is applied to electronic devices used in medicine and surgery to aid in their safe operation; however, IF may occur during use^8^. Since the position of the IF is often out of the surgical field^9^, even the most attentive surgeons might not detect these technical failures (Fig. 1). IF is not only related to endoscopic and robotic surgery^10,11^ but also to the internal medicine field, being used in procedures such as implantable cardioverter-defibrillator treatment^12–14^. The risk of IF lies mainly in reusable instruments; thus, regular evaluation for IF is recommended, and this can be done with the use of a detector^7,15,16^.

The excessive use of reusable instruments can lead to IF, particularly with repetitive passage through trocars, frequent mechanized sterilization processing, and high-voltage usage. However, the relationship between the number of cleanings of reusable products and IF has not been fully investigated. The purpose of this study was to investigate the factors associated with IF in reusable endoscopic instruments.

## Methods

This study was conducted in accordance with the Declaration of Helsinki. Regulatory exemption due to the study’s designation as nonhuman research was obtained from the Ethics Committee of Toyama University Hospital. The study included all reusable endoscopic instruments in the Toyama University Hospital (Toyama, Japan) from June 1st to June 31, 2017.

### Insulation failure check

IF of an instrument was defined as a break or defect confirmed visually or by an insulation tester (DIATEG professional insulation tester, Entrhal Medical GmbH, Straelen, Germany) in the insulation coating of the instrument.

Our hospital staff performed routine visual checks for detectable defects in the insulation coating of reusable instruments. All reusable instruments underwent hand washing of visible stains, followed by mechanized sterilization, and the cleaning numbers were recorded.

**Figure 2:**
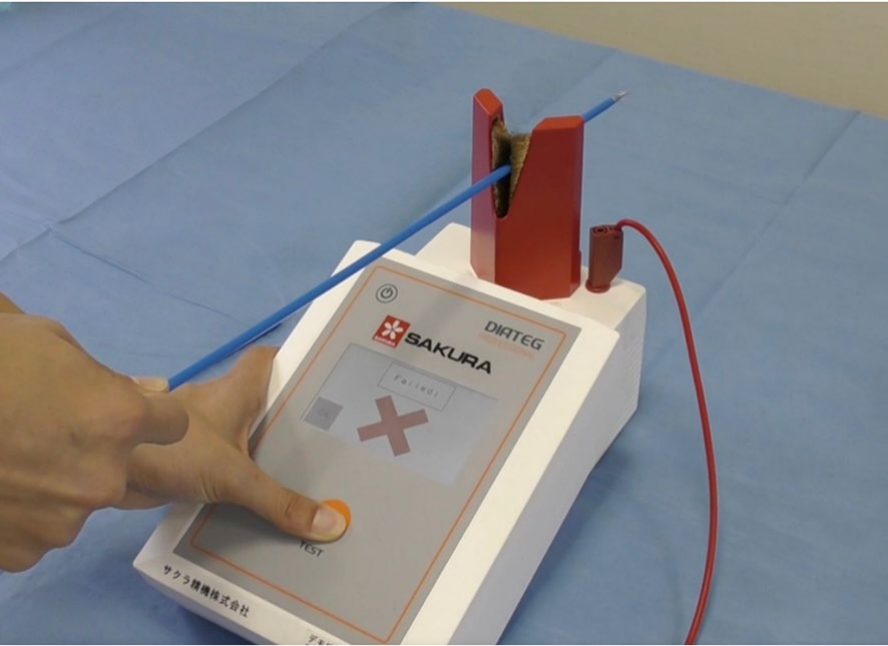
Detecting insulation failure using a DIATEG professional insulation tester.

In this study, IF assessment was performed before sterilization. Firstly, a visual inspection was performed, and then all reusable endoscopic instruments from a variety of surgical specialties were tested for IF during the study using a DIATEG professional insulation tester (Entrhal Medical GmbH, Straelen, Germany) (Fig. 2). Single-use instruments, robotics instruments, cables, and instruments with visually detectable defects were excluded. We followed a previously reported protocol^15^. After the visual inspection, the instrument was connected to the IF detector and the appropriate voltage was selected, 4 kV for monopolar devices and 2 kV for bipolar devices. Voltage selection is important because a low voltage might not detect all defects in monopolar devices, and excessive voltage may damage bipolar tools. Then, the instrument was moved along a metal electrode and an alarm sound alerted the user of an IF. In the case of IF detection, the instrument, location of the defect, and the number of cleanings were recorded. The location of the defect was specified for each instrument and was defined as the distance (cm) from the working tip to the defect/IF.

### Cleaning and sterilization methods

For reusable surgical instruments, cleaning and sterilization were carried out by dedicated staff members based on the guidelines in effect at that time^17–21^. The procedure was as follows:

1. An anticoagulant agent was sprayed on the instrument after surgery. During the cleaning process, each part was disassembled, and, while checking for any damage, blood contamination was removed using running water and brushing. The instrument parts were then soaked in an immersion tank with a neutral enzyme detergent for 30 min, followed by rinsing with running water. The parts were then set in the cleaning device.
2. Machine washing was primarily performed using a vacuum boiling-type washer-disinfector, using a neutral enzyme detergent.
3. After cleaning, the instruments were promptly dried.
4. After drying, a visual inspection was repeated to ensure there was no contamination or damage. The instruments were then lubricated, assembled, and tested before proceeding to the sterilization process.
5. Sterilization was carried out using the appropriate methods for the instruments, such as high-pressure steam (autoclave), ethylene oxide gas, or plasma sterilization. The instruments were wrapped using sterilization containers, non-woven fabric, or sterilization bags, as necessary.
6. After sterilization, the sterilized instruments were stored on shelves dedicated to sterilized items in a designated storage room for sterilized materials for each department.

### Surgical procedures (Table 1).

**Table 1:** IF and surgical use.

Variables	Plastic port	Wound retractor	Monopolar device	Advanced energy device	Number of checked instruments (n)	IF (%)	Distance from the tip to the IF (cm), median [IQR]	Period of use (year), median [IQR]	Number of cleanings (n), median [IQR]
Total					69	8 (11.6)	5 [3–5]	7 [6–8]	261 [211–320]
Gastroenterology	Single	−	++	+	28	4 (14.3)	5 [5–8]	7 [7, 8]	281 [281–350]
Gynecology	Single	–	++	+	20	3 (15.0)	3 [2, 3]	6 [6–8]	261 [233–261]
Urology	Single	–	+	+	12	1 (8.3)	5	8	337
Thoracic	Reuse	+	–	++	9	0 (0)	–	–	–

The number of + indicates frequency.

*IF* insulation failure, *IQR* interquartile range.

#### 1. Gastrointestinal surgery and gynecology

Minimally invasive procedures were performed using multiple single-use plastic ports. The primary technique utilized reusable forceps with monopolar energy devices. However, some surgeons also used advanced energy devices^5^.

#### 2. Urology

Minimally invasive procedures were performed using multiple single-use plastic ports. Approximately half of the techniques employed reusable forceps with monopolar energy devices, while the other half utilized advanced energy devices^5^.

#### 3. Thoracic surgery

Minimally invasive procedures were performed using multiple reusable plastic ports and a wound retractor. Advanced-energy devices were mainly used during the procedures, while techniques involving reusable forceps with monopolar energy devices were not employed for thoracic surgery.

### Variables and assessments

The following parameters were recorded: clinical department using endoscopic instruments (gastrointestinal surgery, gynecology, urology, and thoracic surgery), detected IF, distance from the tip to the IF, period of use, number of cleanings, and manufacturer details.

### Data management and statistical analysis

The number of cleanings and duration of use of the IF and intact instruments were compared. Continuous variables are presented as median with interquartile range for non-normally distributed data. Categorical variables are presented as n (%). For the univariate analysis, intergroup differences were evaluated using the non-parametric Wilcoxon rank-sum test. Statistical significance was defined as *P* < 0.05. All reported *P* values were two-sided. All statistical analyses were performed using JMP version 16.0 (SAS Institute Inc., Cary, NC, USA).

## Results

A total of 69 endoscopic surgical instruments were included in our study. Of these, 28 were used in gastrointestinal surgery, 20 in gynecology, 12 in urology, and 9 in thoracic surgery. IF was found in 8 of the 69 instruments (11.6%) (Table 1). No visual defects were found. The number of IFs per clinical specialty were as follows: 4 of 28 gastrointestinal devices (14.3%), 3 of 20 gynecological devices (15.0%), 1 of 12 urological devices (8.3%), and none of the nine thoracic surgery devices (0%). There was only one insulation defect per instrument. The overall median distance from the tip to the defect was 5 cm (3–5 cm), and the medians by clinical specialty were as follows: gastrointestinal, 5 (5–8) cm; gynecology 3 (2–3) cm; and urology 5 cm.

In the comparison of the IF and the intact instruments, the period of use [7 years (6–8 years) versus 7 years (4–8 years), P = 0.90] and the number of cleanings [281 (261–323) versus 261 (179–320), P = 0.27] showed no significant difference between the instruments (Table 2). The period of use in each specialty was 7 years (7–8 years) for gastrointestinal surgery, 6 years (6–8 years) for gynecology, and 8 years for urology. The number of cleanings was 281 (281–350) in gastrointestinal, 261 (233–261) in gynecological, and 337 in urological devices. The IF instruments were manufactured by three different companies, but six of eight (75.0%) were from the same company.

**Table 2:** Number of cleanings and period of use.

Variables	IF instruments (n = 8)	Intact instruments (n = 61)	P-value
Number of washes (n), median [IQR]	281 [261–323]	261 [179–320]	0.27
Use period (year), median [IQR]	7 [6–8]	7 [4–8]	0.90

*IF* insulation failure, *IQR* interquartile range.

## Discussion

IF of surgical instruments is caused by excessive reuse of instruments, particularly with repetitive passage through trocars, frequent mechanized sterilization processing, and high-voltage applications. The problem that surgeons face is that IF is not always visible, and for this reason regular inspections are recommended using a dedicated detector^7,15,16^. The frequency of IF varies from device to device (Table 3). Yazdani et al. reported an overall IF prevalence of 27% in laparoscopic instruments^22^. In another report, this value was as high as 81.7%^11^. Therefore, regular inspections should be standard procedure at hospitals; however, only a few facilities actually carry out regular inspections. Additionally, the less visible the damage, the higher the current density is, which may make adverse events more likely^1,5^.

**Table 3:** Instruments with IF.

Instrument	Prevalence (%)
Non-laparoscopic	37.5
Monopolar instruments	71.4
Bipolar instruments	62.7–71.4
Laparoscopic	13.1–37.0
Reusable	18.8
Disposable	3.3
Monopolar instruments	6.2
Bipolar instruments	7.7
Robotic	2.6–81.7
Monopolar instruments	71
Bipolar instruments	93
ICD leads	
Riata leads (non-optim)	0.21
Recalled models	0.28–1.14
Biotronik leads	4

*IF* insulation failure, *ICD* implantable cardioverter-defibrillator.

In this study, we examined the relationship between the number of cleanings and IF but found no statistically significance. Reusable products are sold for long-term use, and, in fact, our results showed that long-term use was not always related to IF. When appropriate cleaning is performed, the IF in reused instruments is thought to be influenced by whether the equipment is energized, rather than the duration of use. Unlike advanced bipolar and ultrasonic energy devices, which affect the tissue between the tips of the device, monopolar devices conduct electricity between the active and dispersive electrodes, and this difference in mechanism can lead to IF^1,5^. The use of high voltages increased the risk of insulation damage and capacitive coupling; as such, the operator should understand these restrictions. In a survey of members of the American College of Surgeons, 18% of surgeons had experienced IF or a capacitive coupling injury, and 54% knew a colleague who had a stray electrical burn^4^. Capacitance is defined as stored electrical charge when two conductors are separated by a nonconductive dielectric, also called an insulator^23^. Capacitive coupling occurs when the circuit is completed through the dielectric. Charge will then be stored in the capacitor until either the generator is deactivated or a pathway to complete the circuit is achieved. By its very nature, capacitive coupling can occur only with the use of monopolar instrumentation, and it is not a risk in bipolar instruments because current passes only between the two tips of the active electrodes.

An endoscopic instrument is divided into four zones: near the tip (Zone 1), from the port to the tip (Zone 2), the part passing through the port (Zone 3), and near the operator's hand (Zone 4)^6^. The surgeon’s attention is focused in the Zone 1, and the surgeon expects 100% of the electrosurgical energy to be delivered only to the tissue in that zone. However, the current may be passed inadvertently into the Zones 2–4. Although the sites of IF were in Zones 2 or 3^6^, those of gynecology were more proximal compared to the others. Procedure, port, and the load on the port may be different depending on the specialty. In this study, it was not possible to determine the differences in ports related to the frequency of IF, as various ports were used. However, since there was no IF detected in the thoracic surgery equipment, it can be inferred that wound retractors may be less prone to damage the devices, in line with a previous report^9^.

In this study, 75% of the instruments showing IF were from one company. Since this research did not involve continuous monitoring of the IF from the beginning of product use, and there is a possibility that insulation may have been already improved, it was deemed unfair to disclose the name of the company. The purpose of this research was not to identify defects in a specific product, but rather to investigate the relationship between IF caused by usage and the duration of use. The insulation material applied to laparoscopic instruments is typically a heat shrink material made from a variety of compounds including polyvinylidene fluoride, polyethylene, and polyvinyl chloride. Most laparoscopic devices have an insulation layer that is at least 0.008 cm thick^9^. Therefore, product differences in insulators may have affected the results. Furthermore, a prospective study of differences in IF frequency between robotic surgery and laparoscopic surgery equipment^11^ reported that product differences may have more impact on IF than usage.

We examined the relationship between the number of cleanings and IF of different instruments used in endoscopic surgery. Cleaning methods and frequency have been previously reported as the causes of IF^16^; however, it has been found that, even after long-term use, IF may not occur. Therefore, we considered the differences in the instrument manufacturer and the usage methods to be a bigger influence on IF than the cleaning methods and usage periods.

### Limitations

There are several limitations to this study; it was a single-facility study, and the number of measurements were limited. Since the results were based on a single measurement and not on continuous inspection, it is unknown when the devices were damaged. However, visual inspections were performed regularly, and the instruments were used for long periods of time without causing adverse events. At the time of the study, robotic surgery had just been introduced at our hospital and, as such, our study did not consider robotic instruments.

## Conclusions

Cleaning methods and usage period may have a lower impact on IF compared to the usage methods. The use of reusable forceps as a monopolar device was found to pose a higher risk, requiring regular assessments.

## Data Availability

The datasets used and/or analyzed during the current study are available from the corresponding author on reasonable request.
